# Vitamin D status among infants and children in Shanghai, China: A hospital‐based study

**DOI:** 10.1002/fsn3.3292

**Published:** 2023-03-08

**Authors:** Ying Wu, Fang Wang, Aiguo Li, Jiangfang Gao, Bosheng Li, Huiming Sheng, Jun Ma, Xiang‐Peng Liao

**Affiliations:** ^1^ Department of Pediatrics, Tongren Hospital Shanghai Jiao Tong University School of Medicine Shanghai China; ^2^ Department of Laboratory Medicine, Tongren Hospital Shanghai Jiao Tong University School of Medicine Shanghai China; ^3^ Center for Community Health Care Shanghai Jiao Tong University China Hospital Development Institute Shanghai China

**Keywords:** 25(OH)D, children, deficiency, infants, insufficiency, vitamin D

## Abstract

The variation in vitamin D status is still unclear. We aim to describe the vitamin D status among healthy infants and children in Shanghai (31° N latitude), one of the largest cities in China. We conducted a hospital‐based, 2‐year retrospective observational study and recruited children for health examination at the Tongren Hospital affiliated with Shanghai Jiao Tong University School of Medicine from January 2019 to December 2020. Serum 25‐hydroxyvitamin D (25(OH)D) levels were measured using an enzyme‐linked immunosorbent assay. A total of 6164 children aged 0–11 years were included. Of these, 94.4% of the serum 25(OH)D measurements at first assessment were within the range of 12–50 ng/mL. The median 25(OH)D level was 31.3 (IQR 25.6, 38.1) ng/mL, the percentages of 25(OH)D < 20 ng/mL and 25(OH)D < 30 ng/mL were 10.0% and 43.8%, respectively. Low vitamin D status (deficiency and insufficiency) differed significantly by age group (infants, toddlers, preschoolers, and schoolers) and seasonality (all *p* < .001), but not by gender. For the sub‐group (*n* = 855) of children with repeated assessments, their low 25(OH)D levels increased significantly whether after about a 7‐month (*n* = 351) or 12‐month (*n* = 504) interval, and the increments of median 25(OH)D levels were 8.1 ng/mL and 2.1 ng/mL respectively (*p* < .001). This study documents the vitamin D status in Shanghai, showing that low vitamin D status is common in infants and children and suggesting that the assessment of 25(OH)D level is necessary for individuals who are at risk for deficiency or excess.

## Graphical abstract

This 2‐year observational study assessed serum 25(OH)D levels in 6164 children aged 0–11 years in Shanghai, China. The cross‐sectional and longitudinal data documents the vitamin D status, showing that the prevalence of low vitamin D status was common among infants and children, and the situation was more severe in winter than in other seasons, in school‐age children than in infants, toddlers, and preschoolers. The study suggests that repeated 25(OH)D assessments at about a 7‐month interval, compared with a 12‐month interval, maybe a more effective way to assist in optimizing vitamin D status for those with low vitamin D status.

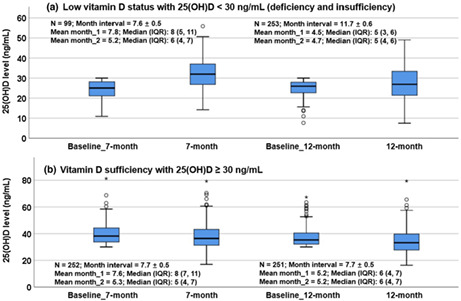

## 1 BACKGROUND

There has been an increasing focus on vitamin D status in the past two decades because vitamin D plays a multifaceted role in the human body. Beyond maintaining skeletal health, research suggests that vitamin D affects many other cellular functions such as cell growth, neuromuscular, immune function, and energy homeostasis (Bouillon et al., 2014; Mailhot & White, 2020; Mayne & Burne, 2019).

However, there is no consensus on optimal serum 25‐hydroxyvitamin D (25(OH)D) concentrations in adults and children. The levels of serum 25(OH)D used to define vitamin D status for different populations differ by organizations and areas, and sometimes the terminology “low vitamin D status” is used for describing deficiency or insufficiency of vitamin D status (Binkley et al., 2010). An expert committee of the Food and Nutrition Board (FNB) at the National Academies of Sciences, Engineering, and Medicine (NASEM) recommended that the cutoffs of serum 25(OH)D concentrations to define vitamin D deficiency (VDD), vitamin D insufficiency (VDI), and vitamin D sufficiency (VDS) be less than 12 ng/mL (30 nmol/L), 12–20 ng/mL (30–50 nmol/L), and 20 ng/mL (50 nmol/L) or more, respectively (Ross et al., 2011); and these cutoffs were used by a global consensus for the prevention and management of nutritional rickets (Munns et al., 2016). In contrast, the Endocrine Society recommended that, to maximize the impact of vitamin D on health in clinical practice, cutoffs of serum 25(OH)D concentrations to define VDD, VDI, and VDS be less than 20 ng/mL (50 nmol/L), 20–30 ng/mL (50–75 nmol/L), and 30 ng/mL (75 nmol/L) or more, respectively (Holick et al., 2011). A recent French expert consensus also suggested a 25(OH)D level of above 30 ng/mL (>75 nmol/L) to avoid any mineralization defects and seasonal variability in general pediatric populations (Bacchetta et al., 2022). The 25(OH)D level associated with toxicity, a rare but potentially harmful side effect of vitamin D treatment, is typically above 150 ng/mL (375 nmol/L), which is well above the level considered to be sufficient (US Preventive Services Task Force et al., 2021; Taylor & Davies, 2018); but has been observed in general pediatric populations when 25(OH)D levels are above 80 ng/mL (200 nmol/L) (Vogiatzi et al., 2014). The FNB cautions against maintaining 25(OH)D levels above 50 ng/mL (125 nmol/L) to avoid potential adverse side effects (Ross et al., 2011). Furthermore, to ensure a large safety margin, the upper limit of 25(OH)D level in general pediatric populations recommended is 100 ng/mL (250 nmol/L) or less by a global consensus (Munns et al., 2016), or more prudently below 60 ng/mL (<150 nmol/L) as suggested by the Canadian Pediatric Society (Vitamin D supplementation: Recommendations for Canadian mothers and infants, 2007) and the French expert consensus (Bacchetta et al., 2022).

A recent review reported the prevalence of VDD worldwide (Amrein et al., 2020). Estimates of the prevalence of 25(OH)D < 20 ng/mL were 24% in the US (Schleicher et al., 2016), 37% in Canada (Sarafin et al., 2015), and 40% in Europe (Cashman et al., 2016). In mainland China, low vitamin D status is now a public health concern requiring multiple approaches to prevention and management (Zhang et al., 2013); and another report focused on pediatric population showed that the prevalence of 25(OH)D < 20 ng/mL was 22.6% among children and adolescents (Yang et al., 2020). In Shanghai, one of the biggest cities in China with a population of more than 20 million in 2021, the reports showed that the prevalence of 25(OH)D < 20 ng/mL was 43% in adults aged 65–95 years (Cheng et al., 2017), 72.5% in pregnant women (Yang, Jing, et al., 2021), and 36.3% in newborns (Yu et al., 2014). However, to our knowledge, there is no study on the vitamin D status of infants and children in Shanghai.

Several factors contribute to the variation in vitamin D status, including age, skin color, season, diet and nutrition, ethnic and cultural background, lifestyle, and underlying disease (Amrein et al., 2020; Cashman et al., 2016; Holick et al., 2011; US Preventive Services Task Force et al., 2021). There is contradictory evidence on the effect of urban living on vitamin D status, but it is a concern given the increasing numbers of people worldwide residing in urban areas. For example, a study from India showed that older adult residents had lower 25(OH)D levels in urban compared with rural communities (Harinarayan et al., 2008). In contrast, higher 25(OH)D levels were reported for urban compared with rural dwellers in Ireland and China (Fang et al., 2018; Griffin et al., 2020).

Given the limited knowledge on the vitamin D status of infants and children in Shanghai, the study aimed to investigate the vitamin D status of children aged 0–11 years in this large and highly urbanized city. Also, currently, there are few longitudinal data on vitamin D status in pediatric populations, so repeated 25(OH)D measurements in the sub‐group were also included in this study.

## 2 METHODS

### 2.1 Design and setting

This study used a retrospective observational design. The study was conducted from January 2019 to December 2020, at the Tongren Hospital affiliated with Shanghai Jiao Tong University School of Medicine in Shanghai, China. The hospital is a regional tertiary‐care central hospital located in Changning district and serves a population of about 1.5 million. The child healthcare clinic of the hospital serves children from three districts in urban Shanghai (Changning, Minhang, and Putuo).

### 2.2 Study population

Healthy children aged 0–11 years were recruited during their regular health examination visits to child healthcare clinic in the hospital. The healthcare providers informed guardians of procedures in the child health examination, offering the choice of serum 25(OH)D test. Since vitamin D plays multifactorial roles in diseases and health, a choice of reassessing the 25(OH)D levels after time intervals was offered to the guardians, and the rationale behind the reassessing included request of guardians, previous vitamin D status, interventions of vitamin D supplementation, or sun exposure. Informed consent was obtained from guardians involved in the study. Participants were excluded if they had any known underlying diseases that affect vitamin D metabolisms, such as skeletal diseases, genetic syndromes, malabsorptive disorders, or abnormal liver or renal function.

### 2.3 Sample collection and assessment of vitamin D status

A small blood sample (40 μL) was collected from each child participant using a finger stick. Serum samples were stored in a refrigerator at 4°C and tested within 3 days. Serum 25(OH)D concentration was measured using an enzyme‐linked immunosorbent assay following the manufacturer's instructions (IDS Ltd.). The inter‐assay and intra‐assay coefficients of variation were <10%, respectively.

### 2.4 Statistical analyses

Participants were stratified by gender, age, and seasonality. Age was divided into four groups: infants (0–1 year), toddlers (1–3 years), preschool children (3–6 years), and school‐age children (6–11 years). Seasonality for blood sample collection was defined as four groups: spring (March, April, May), summer (June, July, August), autumn (September, October, November), and winter (December, January, February).

To build on the literature, we used two sets of recommended cutoffs for vitamin D status using serum 25(OH)D in our analyses. First, and following the FNB recommendation (Rosen et al., 2012), the cutoffs used for VDD, VDI, and VDS were serum 25(OH)D levels (ng/mL) < 12, 12–20, and ≥20, respectively. Second, and following the Endocrine Society guidelines (Holick et al., 2011), the cutoffs used for VDD, VDI, and VDS were serum 25(OH)D levels (ng/mL) < 20, 20–30, and ≥30, respectively.

Analyses of the serum 25(OH)D measurements showed a skewed distribution; the data were therefore described using median and percentile values. Percentages (%) were reported for categorical variables. Stratified analyses were conducted by gender, age group, seasonality, 25(OH)D level, and time interval. The nonparametric Chi‐square test was used to compare differences between groups, and the Wilcoxon signed‐rank test was used to compare the repeated measurements. The association between gender, age, and seasonality with low vitamin D status was determined through multinomial logistic regression. The results were considered to be statistically significant when the 2‐tailed *p*‐value was <.05. Statistical analyses were performed using SPSS 26.0 (IBM Corp).

### 2.5 Ethical considerations

The study was conducted in accordance with the Declaration of Helsinki, and approved by the Institutional Review Board of Tongren Hospital affiliated with Shanghai Jiao Tong University School of Medicine.

## 3 RESULTS

During this 2‐year observational span, we recruited 6164 participants (3185 boys and 2979 girls) aged 0–11 years for the study. The participants had at least an assessment of serum 25(OH)D level. Of them, one sub‐group of 351 participants had twice assessments at about a 7‐month interval, and the other sub‐group of 504 participants had twice assessments at about a 12‐month interval.

### 3.1 Serum 25(OH)D levels at first assessment by gender, age group, and seasonality

**FIGURE 1:**
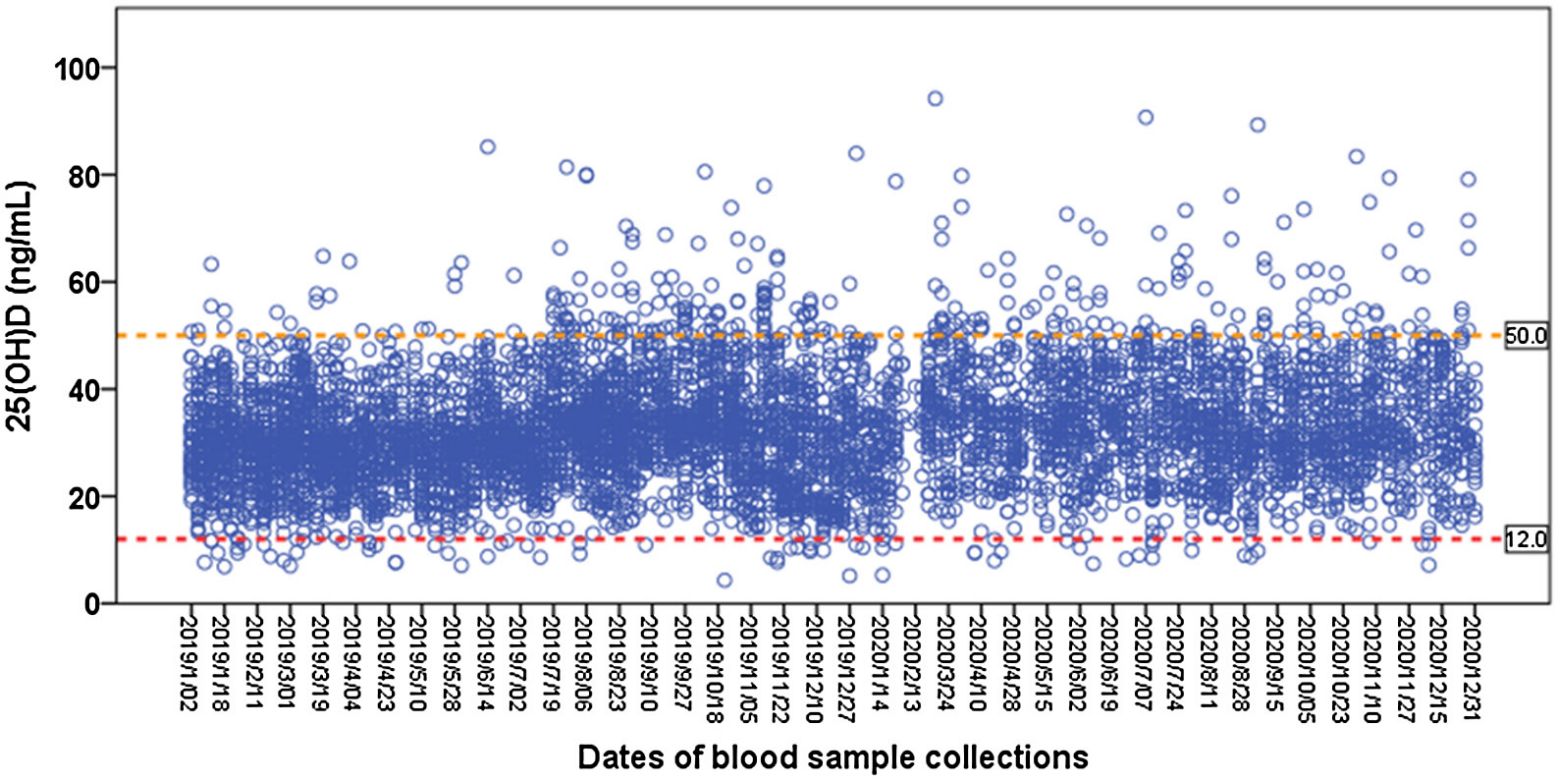
Scatter distributions of serum 25(OH)D levels by dates of blood sample collections. The 25(OH)D measurements above the upper line (50 ng/mL) account for 4.4%. The 25(OH)D measurements below the lower line (12 ng/mL) account for 1.2%, which are considered deficient according to the Food and Nutrition Board (FNB).

Of the 6164 participants with the first measurement, the median 25(OH)D level was 31.3 (IQR 25.6, 38.1) ng/mL; and the 25(OH)D levels ranged from 4.3 ng/mL to 94.2 ng/mL. The majority (94.4%) of the 25(OH)D measurements were within the range of 12–50 ng/mL, and 4.4% (272/6164) of the measurements were higher than 50 ng/mL (Figure 1). The frequency distribution of 25(OH)D measurements is shown in Figure S1.

The 25(OH)D levels by gender, age group, seasonality, and corresponding percentiles are detailed in Table 1. There was no significant difference in 25(OH)D levels between boys and girls, but there was a significant difference across the four age groups (*p* < .001), with the preschoolers having the highest levels and the school‐age children having the lowest levels (median: 32.6 ng/mL vs. 30.0 ng/mL). Meanwhile, the 25(OH)D levels in summer and autumn were significantly higher than in spring and winter, respectively (all *p* < .001). The difference in median 25(OH)D levels between summer and winter was 2.6 ng/mL. Children reached the highest levels in September (median: 33.5 ng/mL), and the lowest levels in February (median: 28.7 ng/mL) (Figure S2).

**TABLE 1:** Serum 25(OH)D levels (ng/mL) by gender, age group, and seasonality (*N* = 6164).

Variables	*N*	Mean	Minimum	Maximum	Percentiles						
					5	10	25	50	75	95	97
All	6164	32.3	4.3	94.2	17.4	20.0	25.6	31.3	38.1	49.4	52.2
Gender*											
Boys	3185	32.3	6.9	90.7	17.5	19.9	25.7	31.4	37.9	49.2	52.5
Girls	2979	32.3	4.3	94.2	17.3	20.1	25.4	31.3	38.4	49.5	52.2
Age (years)**											
0–1	1460	33.2	7.0	84.0	18.9	22.0	26.9	31.9	38.8	45.6	52.7
1–3	1973	30.3	4.3	83.4	17.5	19.3	24.2	29.4	34.9	42.4	49.4
3–6	2336	33.3	5.3	94.2	16.9	19.9	26.3	32.6	40.0	47.0	53.8
6–11	395	33.4	8.5	76.0	14.2	18.2	25.1	32.9	41.6	48.6	55.2
Seasonality***											
Spring	1599	31.3	7.0	94.2	17.4	19.5	24.9	30.3	36.9	48.4	51.2
Summer	1777	33.1	7.4	90.7	19.0	21.3	26.5	32.1	38.7	49.2	52.2
Autumn	1521	33.9	4.3	89.3	18.2	21.3	27.1	32.6	40.2	51.9	56.1
Winter	1267	30.5	5.2	84.0	15.8	18.0	23.5	30.0	36.4	48.0	49.8

* The 25(OH)D levels had no significantly difference between gender (*p* = .99).

** The 25(OH)D levels were significantly different in children of different age groups (*p* < .001).

*** The 25(OH)D levels were significantly different among different seasons (*p* < .001).

### 3.2 Vitamin D status at first assessment by gender, age group, and seasonality

At first assessment, the percentages of 25(OH)D < 20 ng/mL and 25(OH)D < 30 ng/mL were 10.0% and 43.8%, respectively. Using the FNB cutoffs, the prevalence of VDD and VDI were 1.2% and 8.8%, respectively; using the Endocrine Society cutoffs, the prevalence of VDD and VDI were 10.0% and 33.8%, respectively (Table 2).

**TABLE 2:** Vitamin D status according to different cutoffs by gender, age group, and seasonality (*N* = 6164).^a^

	Food and nutrition board				Endocrine society			
Variables	VDD	VDI	VDS	*p*‐Value	VDD	VDI	VDS	*p*‐Value
All	73 (1.2)	545 (8.8)	5546 (90.0)		618 (10.0)	2083 (33.8)	3463 (56.2)	
Gender								
Boys	35 (1.1)	290 (9.1)	2860 (89.8)	.62	325 (10.2)	1053 (33.1)	1807 (56.7)	.45
Girls	73 (1.2)	255 (8.6)	2686 (90.2)		293 (9.8)	1030 (34.6)	1656 (55.6)	
Age (years)								
0–1	7 (0.5)	90 (6.2)	1363 (93.4)	<.001	97 (6.6)	476 (32.6)	887 (60.8)	<.001
1–3	12 (0.6)	212 (10.7)	1749 (88.6)		224 (11.4)	837 (42.4)	912 (46.2)	
3–6	44 (1.9)	195 (8.3)	2097 (89.8)		239 (10.2)	680 (29.1)	1417 (60.7)	
6–11	10 (2.5)	48 (12.2)	337 (85.3)		58 (14.7)	90 (22.8)	247 (62.5)	
Seasonality								
Spring	20 (1.3)	153 (9.6)	1426 (89.2)	<.001	173 (10.8)	612 (38.3)	814 (50.9)	<.001
Summer	17 (1.0)	107 (6.0)	1653 (93.0)		124 (7.0)	576 (32.4)	1077 (60.6)	
Autumn	11 (0.7)	115 (7.6)	1395 (91.7)		126 (8.3)	447 (29.4)	948 (62.3)	
Winter	25 (2.0)	170 (13.4)	1072 (84.6)		195 (15.4)	448 (35.4)	624 (49.3)	

^a^ Values are expressed as n (%), and the data were analyzed with the Chi‐square test.

There was no significant difference in vitamin D status between boys and girls, but there were significant differences across age groups and seasons (all *p* < .001). Overall, the prevalence of 25(OH)D < 20 ng/mL was lower in the infants than in other age groups, and the prevalence of VDD was higher among school‐age children. The prevalence of 25(OH)D < 30 ng/mL was 50.7% in winter and 49.1% in spring, which was higher than in summer (39.4%) and autumn (37.7%). The prevalence of low vitamin D status by age group and seasonality is shown in Figures S3 and S4.

Following the Endocrine Society's definition of vitamin D status, a multinomial logistic regression model also found that children in winter had a higher risk of deficiency (OR 2.36; 95% CI 1.84, 3.02) and insufficiency (OR 1.46; 95% CI 1.24, 1.73) than in autumn (Table S1).

### 3.3 Repeated measurements of serum 25(OH)D levels by time interval

Of the 855 participants with repeated assessments, either at about a 7‐month (7.6 ± 0.5) or 12‐month (11.7 ± 0.6) interval, their 25(OH)D measurements ranged from 7.5 ng/mL to 81.3 ng/mL. The median 25(OH)D levels at the first and second assessments were 31.7 ng/mL and 32.6 ng/mL, respectively, and there was no significant difference across time. The prevalence of 25(OH)D < 30 ng/mL at the first and second assessments was 58.9% (504/855) and 59.1% (505/855), respectively.

Stratified analyses by time interval found that the 25(OH)D levels at the 7‐month interval (*n* = 351) were significantly higher than at the 12‐month interval (*n* = 504) (*p* < .001). The stratified analyses based on the baseline vitamin D status according to the Endocrine Society also found that the 25(OH)D levels in the VDD (*n* = 55) and VDI (*n* = 297) groups improved significantly; but the relatively high 25(OH)D levels in the VDS (*n* = 503) group at baseline lowered significantly over time (all *p* < .001) (Table 3).

**TABLE 3:** Repeated measurements of timing and serum 25(OH)D levels in sub‐groups^a^ (*N* = 855).

		Measurement_1 (baseline)				Measurement_2 (follow‐up)				*p*‐Value^c^
		Month^b^		25(OH)D (ng/mL)		Month^b^		25(OH)D (ng/mL)		
	*n*	Mean	Median (IQR)	Mean	Median (IQR)	Mean	Median (IQR)	Mean	Median (IQR)	
ALL	855	6.0	6 (4, 8)	32.9	31.7 (26.8, 38.2)	5.1	5 (4, 7)	33.0	32.6 (25.5, 38.8)	.93
Time interval										
7‐month	351	7.7	8 (5, 11)	35.4	34.3 (29.2, 42.0)	5.3	6 (4, 7)	35.9	35.0 (29.4, 41.2)	.56
12‐month	504	4.9	5 (3, 7)	31.2	30.0 (25.9, 35.3)	5.0	5 (4, 7)	31.0	30.0 (23.7, 35.9)	.58
25(OH)D level^d^										
VDD	55	5.9	5 (3, 8)	17.3	18.1 (16.3, 18.7)	5.0	5 (3, 8)	27.1	27.2 (20.8, 33.3)	<.001
VDI	297	5.4	5 (4, 7)	26.1	26.5 (24.1, 28.4)	4.8	5 (4, 7)	29.3	28.6 (22.6, 34.6)	<.001
VDS	503	6.4	7 (4, 8)	38.7	36.7 (32.9, 42.6)	5.3	5 (4, 7)	35.9	34.8 (28.8, 41.4)	<.001

^a^ The participants were with repeated measurements, either at about a 7‐month or 12‐month interval.

^b^ For the comparison, the values of January to December were transformed as numbers 1 to 12, respectively, and the values of months were presented as mean and median (IQR).

^c^ The *p*‐values were based on the repeated measurements of 25(OH)D levels.

^d^ The vitamin D status was classified as VDD (<20 ng/mL), VDI (20–30 ng/mL) VDS (≥30 ng/mL), respectively, according to the Endocrine Society.

**FIGURE 2:**
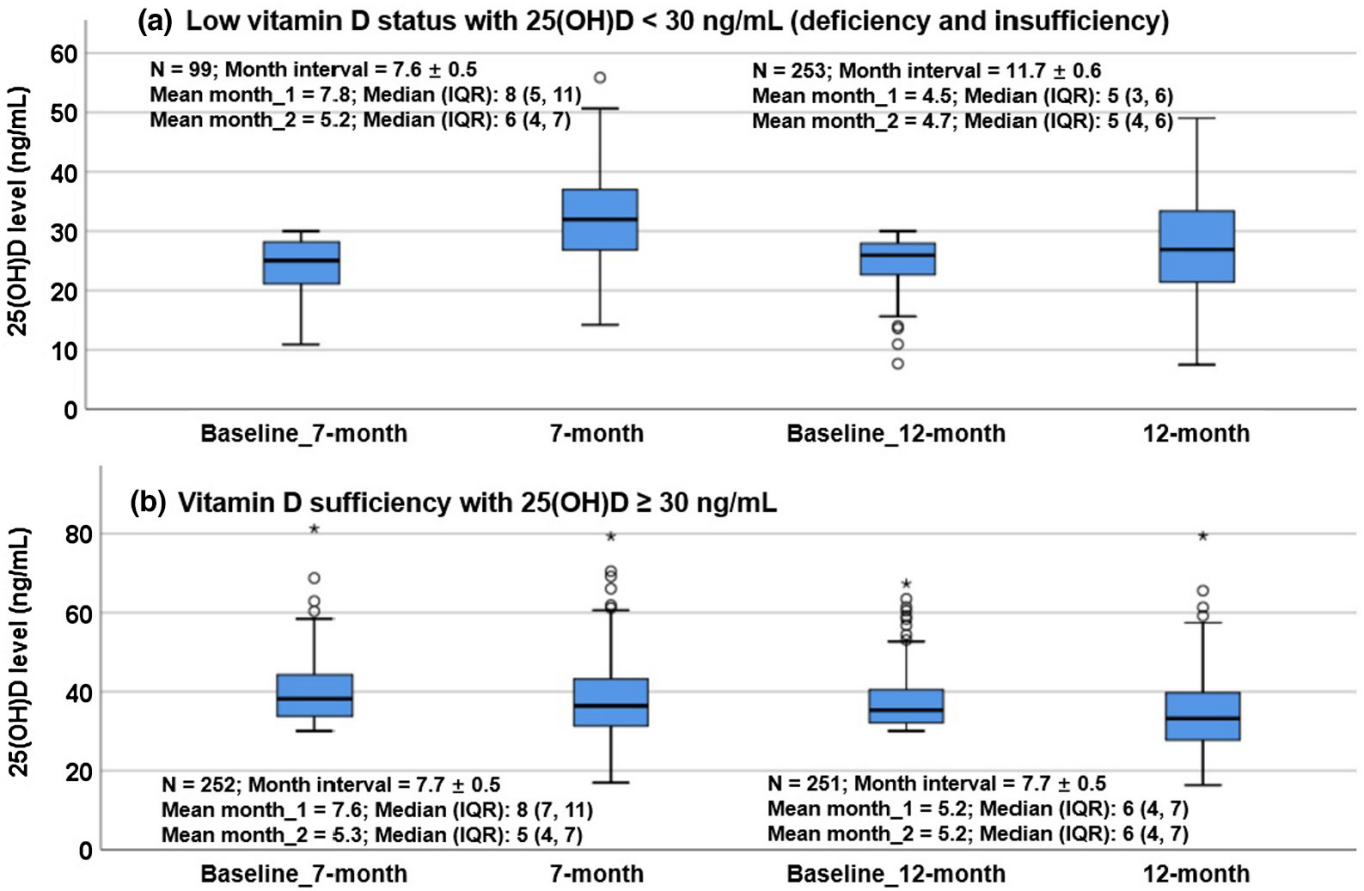
Variations of serum 25(OH)D levels in the participants after repeated assessments. (a) The participants had 25(OH)D < 30 ng/mL at baseline; their median 25(OH)D levels increased significantly after about a 7‐month interval (25.0 ng/mL vs. 32.0 ng/mL), and a 12‐month interval (25.9 ng/mL vs. 26.9 ng/mL), respectively; but the increment of median 25(OH)D level in the 7‐month interval (8.1 ng/mL) was significantly obvious than that in the 12‐month interval (2.1 ng/mL) (all *p* < .001). (b) The participants had 25(OH)D ≥ 30 ng/mL at baseline; their median 25(OH)D levels lowered significantly after a 7‐month interval (38.2 ng/mL vs. 36.4 ng/mL), and a 12‐month interval (35.3 ng/mL vs. 33.2 ng/mL), respectively (all *p* < .001). For the comparison, the values of January to December are transformed as numbers 1–12, respectively, and the values of months are presented as mean and median (IQR). The circle dots represent mildly abnormal values (outside of Q1‐1.5 times IQR or Q3+1.5 times IQR), while asterisk dots indicate extreme abnormal values (outside of Q1‐3 times IQR or Q3+3 times IQR).

Variation in serum 25(OH)D levels after the repeated assessments also found that for participants with 25(OH)D < 30 ng/mL at the baseline, there was a significantly larger increment of change in median 25(OH)D levels at the 7‐month interval than for those at the 12‐month interval (8.1 ng/mL, *n* = 99; vs. 2.1 ng/mL, *n* = 253) (*p* < .001). In contrast, for participants with 25(OH)D ≥ 30 ng/mL at baseline, there was no significant difference in the increment of change in median 25(OH)D levels at the 7‐month and 12‐month intervals (2.4 ng/mL, *n* = 252; vs. 3.7 ng/mL, *n* = 251) (*p* = 0.27) (Figure 2).

## 4 DISCUSSION

We conducted a hospital‐based, 2‐year observational study in a large urban area in China. We examined the vitamin D status in 6164 infants and children aged 0–11 years and found that 90.0% of the participants were serum 25(OH)D ≥ 20 ng/mL, 56.2% of the participants were serum 25(OH)D ≥ 30 ng/mL.

Our data suggest that the 25(OH)D levels in Shanghai are somewhat higher than most reports worldwide. A 25(OH)D level analysis of the National Health and Nutrition Examination Survey 2011–2014 data in the USA found that 7.1% of children aged 1–5 years and 13.7% of children aged 6–11 years were 25(OH)D < 20 ng/mL (Herrick et al., 2019). The Canadian Health Measures Survey reported that 21.8% of children aged 9–13 years were 25(OH)D < 20 ng/mL (Sarafin et al., 2015). Studies from Europe found that about 40% of the children were 25(OH)D < 20 ng/mL (Cashman et al., 2016; Manios et al., 2018). In Asia, the Japan Environment and Children's Study estimated the prevalence of 25(OH)D < 20 ng/mL and 25(OH)D < 30 ng/mL among children aged 2 years were 25.4% and 75.9%, respectively (Yang et al., 2021); Lower vitamin D status is also prevalent in southern Asian countries, such as India where about 40%–70% of infants and over 80% of school‐aged children were 25(OH)D < 20 ng/mL (Kamboj et al., 2018). A report found higher vitamin D status in five countries in Sub‐Saharan Africa in 4509 children aged 0–8 years, where the prevalence of 25(OH)D < 20 ng/mL and 25(OH)D < 30 ng/mL were 7.8% and 44.9% respectively, with the overall median 25(OH)D of 31.0 (IQR 25.4, 37.7) ng/mL (Mogire et al., 2021); and VDD is also more prevalent in northern African countries (Mogire et al., 2020). Studies also reported that the prevalence of 25(OH)D < 20 ng/mL among preschool children was 24% in Mexico (Brito et al., 2013), and 48% in New Zealand (Cairncross et al., 2017).

Consistent with other studies, the reports from China also show variations in vitamin D levels. A study across China reported that the prevalence of 25(OH)D < 20 ng/mL was 22.6% among children and adolescents, with a mean 25(OH)D of 28.8 ng/mL (Yang et al., 2020). In Harbin (latitude 45.8° N), the prevalence of 25(OH)D < 30 ng/mL was 70% in children aged 0–12 years, with the mean 25(OH)D of 24.8 ng/mL (Wei et al., 2018). In Hangzhou (latitude 30.2° N), the prevalence of 25(OH)D < 30 ng/mL was 64% in children aged 2–5 years, with the mean 25(OH)D of 24.8 ng/mL (Chen et al., 2021). However, The middle size city of Huzhou (latitude 30.9° N) reported a higher vitamin D level, where 23.3% of children aged 0–17 years were 25(OH)D < 30 ng/mL with the mean of 25(OH)D of 41.0 ng/mL (Wang et al., 2017). These variations are partly due to sunlight exposure related to latitude and outdoor activity, as well as vitamin D supplementation in daily practice.

Also, consistent with other studies (Cashman et al., 2016; Herrick et al., 2019; Sarafin et al., 2015; Yang et al., 2020; Yang, Jing, et al., 2021), we found significant seasonal and age group variation in vitamin D status. Furthermore, the differences in vitamin D status among age groups are also consistent with the studies in the literature (Herrick et al., 2019; Mogire et al., 2020; Yang et al., 2020). However, we found no significant differences in vitamin D status between boys and girls. Some studies have reported higher vitamin D levels in men than women, which may be related to outdoor activities and religious and cultural practices (Mogire et al., 2020). Meanwhile, although 4.4% (272/6164) of the children in our study were 25(OH)D ≥ 50 ng/mL (maximum 90.7 ng/mL), we found no vitamin D intoxication or reported side effects (Vogiatzi et al., 2014).

A number of factors may have contributed to our finding of relatively higher vitamin D status. Urbanization may have multiple effects on the 25(OH)D levels. Urbanization is commonly linked to the increase in air pollution, limited land space, and lower physical activity in the city (Yang et al., 2018). High‐rise buildings in Shanghai squeeze a limited urban area with a high population density, and environmental pollution undeniably affects Ultraviolet exposure. Meanwhile, the study found that very few children and adolescents in Shanghai showed active lifestyles (Chen et al., 2018). Thus, these factors can adversely affect vitamin D synthesis in the human body. Nevertheless, urbanization can provide residents with the convenience of accessing health services, and the relatively higher socioeconomic status and health awareness of the families around the study hospital (Zhang & Li, 2011), can benefit their health behaviors and child healthcare.

Furthermore, the Chinese Medical Association has recommended vitamin D supplementation, similar to the American Academy of Pediatrics (Wagner & Greer, 2008), and this practice has been implemented for many years in Shanghai. It is almost routine for parents to supply vitamin D to their children, especially infants and toddlers, usually at 400–600 IU/d levels. Also, primary community hospitals in the city recommend vitamin D supplementation for toddlers and preschoolers. Thus, these factors have improved vitamin D status in this study.

This study supports previous research pointing to the need to assess vitamin D status in at‐risk segments of the pediatric population (Munns et al., 2016). For the individuals in the VDS group with repeated measurements, the relatively high median 25(OH)D level at the baseline alleviated after the intervals (36.7 ng/mL vs. 34.8 ng/mL); and this change was partly due to the healthcare providers' concern about the side effects of excessive vitamin D intake, as the individuals' vitamin D status was already sufficient at the first assessment. Moreover, our 7‐month and 12‐month assessments of 25(OH)D show an increase in low vitamin D status; and the variations of serum 25(OH)D levels over time also show that a 7‐month assessment of vitamin D status is more effective than a 12‐month assessment for those with low vitamin D status (Table 3 and Figure 2).

Remarkably, the repeated 25(OH)D assessments at the 7‐month interval in the participants with low vitamin D status showed a larger increment (8.1 ng/mL vs. 2.1 ng/mL) compared with the 12‐month interval. While taking into account the seasonal variation of the serum 25(OH)D levels, this study found that serum median 25(OH)D levels at first assessments peaked in September (median: 33.5 ng/mL) and reached the lowest in February (median: 28.7 ng/mL) with the difference of about 4.7 ng/mL (14%) (Figure S2). Meanwhile, for the group of 7‐month interval with 25(OH)D < 30 ng/mL, the mean month of the first measurements was 7.8, and 63.6% (65/99) of the measurements were within summer and autumn, when the subjects would naturally have a higher 25(OH)D level (Figure S2 and Figure 2). In contrast, the mean month of the second measurement was 5.2, and 44.4% (44/99) of the measurements were within winter and spring, when the subjects would naturally have a lower 25(OH)D level. Thus, the more significant increment of 25(OH)D levels after the 7‐month interval than the 12‐month interval could not be due to seasonal variation. Instead, the healthcare providers could lead in this improvement by guiding the children to optimize vitamin D status with the reference of baseline 25(OH)D levels. In addition, parents of the 7‐month interval group may have more health awareness of vitamin D, be more willing to cooperate with healthcare providers for vitamin D assessment, and more compliant with vitamin D supplementation, or sun exposure.

Further, studies also suggest that vitamin D status can be worse in newborns and adolescents than in toddlers, preschoolers, and schoolers (Cashman et al., 2016; Herrick et al., 2019; Mogire et al., 2020; Yu et al., 2014), or among overweight and obese than normal‐weight children (Turer et al., 2013; US Preventive Services Task Force et al., 2021). Thus, assessing vitamin D status can be more necessary in these individuals, and further study may focus on developing a validated tool for screening based on risk factors in populations. For individuals with vitamin D as supplementary treatment, regular assessments should be a component of treatment.

Strengths of this study include that it is a 2‐year study with cross‐sectional and longitudinal data in one of the biggest cities in China, and represents a large sample size that covered healthy infants and children. Given that there are limited representative data on vitamin D status in pediatric populations, this study could provide public health information and assist future efforts to optimize vitamin D status in pediatric populations. There were some limitations with caution. This study did not collect anthropometric measurements, sociodemographic characteristics, diet and vitamin D supplements, and sunlight exposure. This hospital‐based study does not necessarily take into account the status of the general population aged 0–11 years. However, to our best knowledge, this study involves a large sample of children in the city of Shanghai, and the participants of the health examination were representative of the infants and children in urban Shanghai.

## 5 CONCLUSIONS

This study documents the vitamin D status of children aged 0–11 years in Shanghai and identifies a high prevalence of low vitamin D status. The situation was more evident in winter than in other seasons, in school‐age children than in infants, toddlers, and preschoolers. Meanwhile, our findings support the positive role of assessing 25(OH)D levels for individuals who are at risk for deficiency or excess.

## FUNDING INFORMATION

This research was supported by the Quality‐Balance Research Talent Development Fund of Changning District, Shanghai (CNYZ01), and the Research Talent Fund of Shanghai Tongren Hospital, Shanghai Jiao Tong University School of Medicine (TR202001).

## CONFLICT OF INTEREST STATEMENT

The authors declare that they have no conflicts of interest.

## Supporting information


Data S1.

## Data Availability

The data that support the findings of this study are available from the corresponding author upon reasonable request.
