# Supplementary material for: Vitamin D status among infants and children in Shanghai, China: A hospital‐based study
Source: Food Sci Nutr. 2023 Mar 8;11(6):3111–20. doi: 10.1002/fsn3.3292 (PMC10261785; doi:10.1002/fsn3.3292)
Supplement: Supplementary file 1 — Data S1. [file FSN3-11-3111-s001.docx]

**Supplementary Appendix**


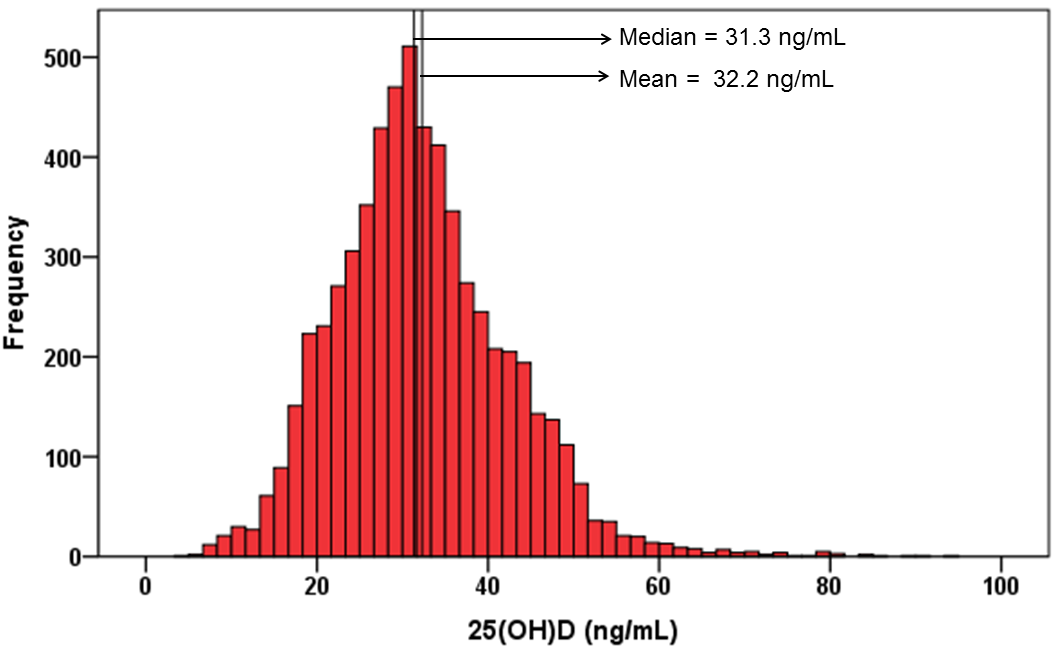


**FIGURE S1** Frequency distributions of serum 25(OH)D measurements in the participants.


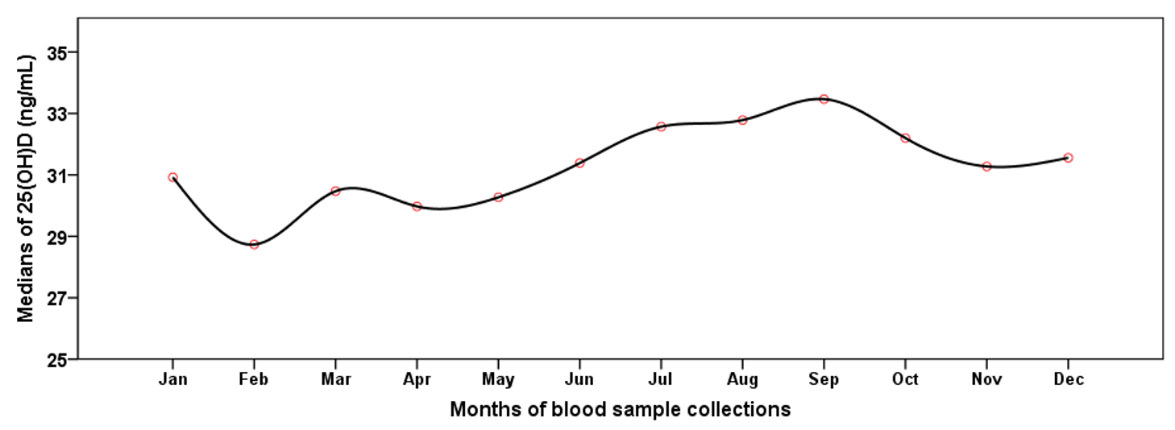


**FIGURE S2** Seasonal variation of the serum 25(OH)D levels in the participants. The difference in serum median 25(OH)D levels between summer and winter was 2.6 ng/mL (8%), and the difference in serum median 25(OH)D levels between September and February was 4.7 ng/mL (14%).


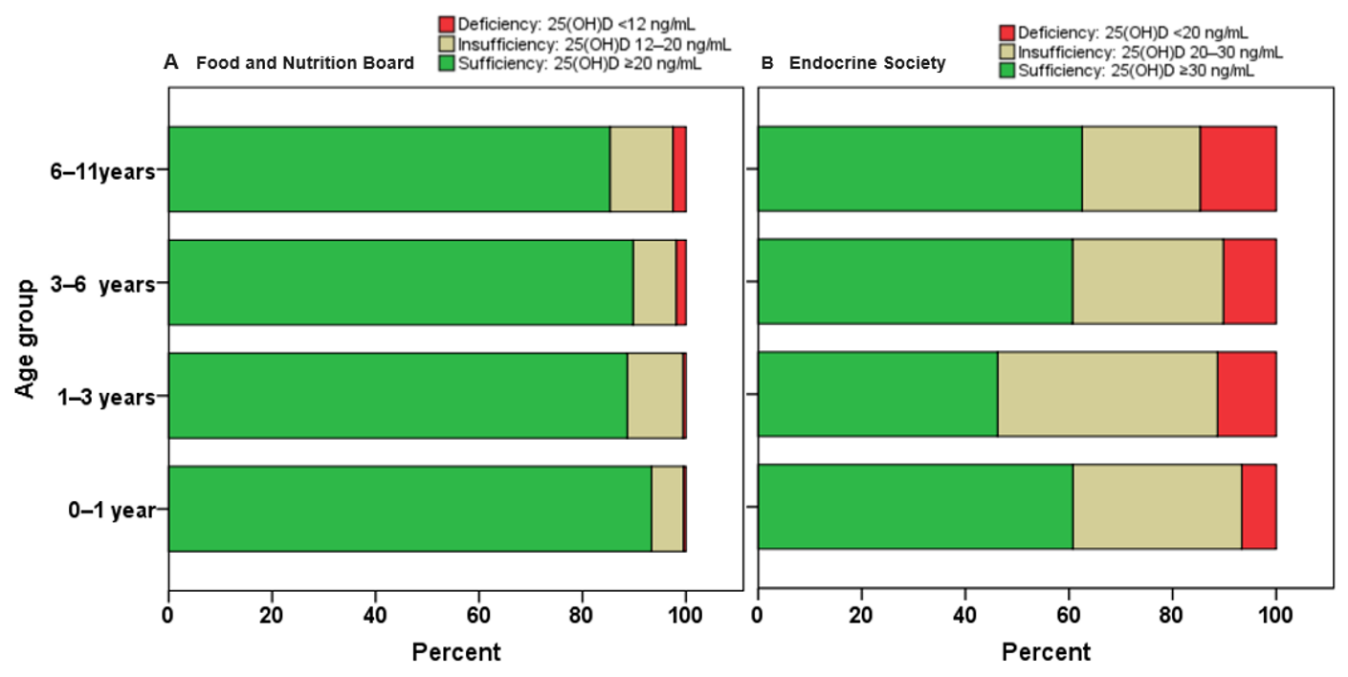


**FIGURE S3** Prevalence of vitamin D status in the participants by age group with different standards. **A**: The prevalence of vitamin D deficiency (VDD) with the cutoff of 25(OH)D < 12 ng/mL (red color) was low, according to the Food and Nutrition Board (FNB). **B:** The prevalence of VDD with the cutoff of 25(OH)D < 20 ng/mL (red color) was most common in school-age students, according to the Endocrine Society.


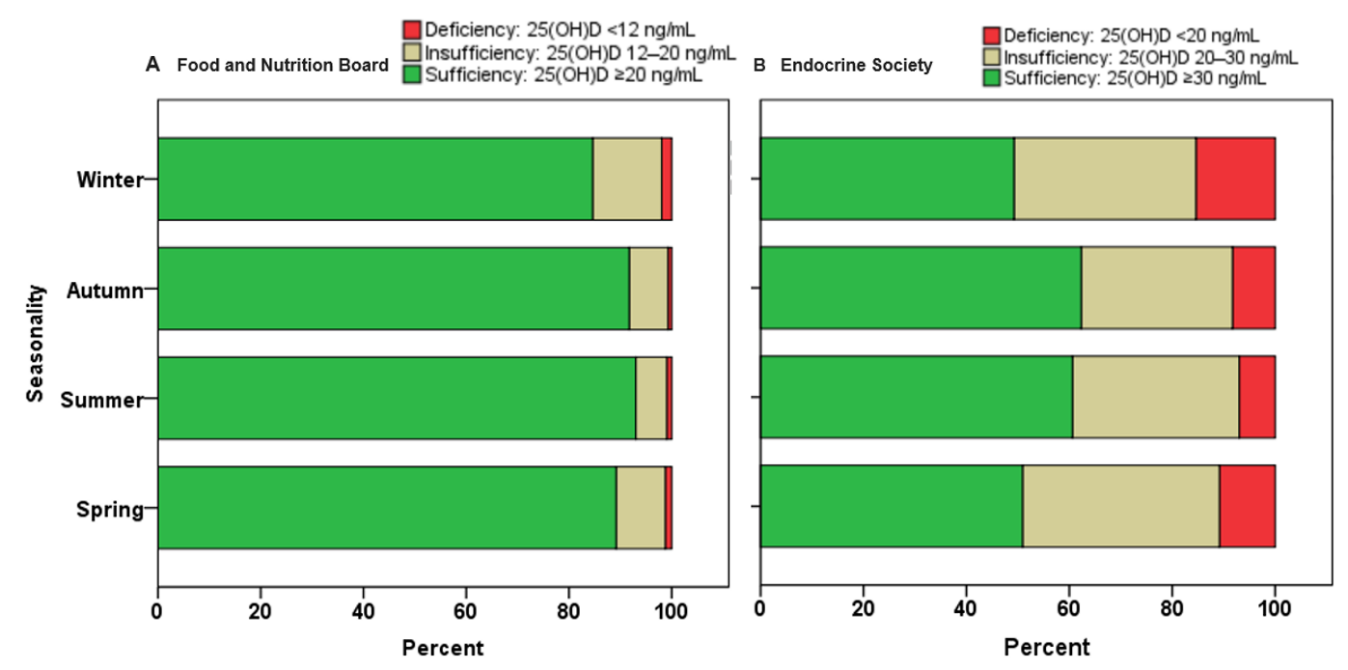


**FIGURE S4** Prevalence of vitamin D status in the participants by seasonality with different standards. **A**: The prevalence of vitamin D deficiency (VDD) with the cutoff of 25(OH)D < 12 ng/mL (red color) was low among different seasons, according to the Food and Nutrition Board (FNB). **B:** The prevalence of VDD with the cutoff of 25(OH)D < 20 ng/mL (red color) was most common in winter, according to the Endocrine Society.

**TABLE S1**  The association of gender, age, and seasonality with low vitamin D status (categorical variable) in single measure* (*N* = 6164)

|  | **VDD (< 20 ng/mL)** | | |  | **VDI (20–30 ng/mL)** | | |
| --- | --- | --- | --- | --- | --- | --- | --- |
| **Variables** | **Beta Coefficients** | **OR (95% CI)** | ***p*-Value** |  | **Beta Coefficients** | **OR (95% CI)** | ***p*-Value** |
| Gender |  |  |  |  |  |  |  |
| Boys | 0.01 | 1.01 (0.85, 1.20) | 0.91 |  | -0.06 | 0.95 (0.85, 1.06) | 0.32 |
| Girls | REF |  |  |  | REF |  |  |
| Age (year) |  |  |  |  |  |  |  |
| 0–1 | REF |  |  |  |  |  |  |
| 1–3 | 0.83 | 2.30 (1.78, 2.98) | <0.001 |  | 0.55 | 1.73 (1.49, 2.00) | <0.001 |
| 3–6 | 0.49 | 1.63 (1.27, 2.10) | <0.001 |  | -0.09 | 0.91 (0.79, 1.05) | 0.20 |
| 6–11 | 0.85 | 2.35 (1.64, 3.36) | <0.001 |  | -0.35 | 0.70 (0.54, 0.92) | 0.01 |
| Seasonality |  |  |  |  |  |  |  |
| Spring | 0.48 | 1.62 (1.26, 2.07) | <0.001 |  | 0.46 | 1.59 (1.36, 1.86) | <0.001 |
| Summer | -0.17 | 0.84 (0.65, 1.10) | 0.21 |  | 0.11 | 1.11 (0.96, 1.30) | 0.17 |
| Autumn | REF |  |  |  |  |  |  |
| Winter | 0.86 | 2.36 (1.84, 3.02) | <0.001 |  | 0.38 | 1.46 (1.24, 1.73) | <0.001 |

*The multinomial logistic regression model includes all variables in this table, and the vitamin D status was classified according to the Endocrine Society.
